# The impact of diagnostic delay on survival in alpha-1-antitrypsin deficiency: results from the Austrian Alpha-1 Lung Registry

**DOI:** 10.1186/s12931-023-02338-0

**Published:** 2023-01-27

**Authors:** Tobias Meischl, Karin Schmid-Scherzer, Florian Vafai-Tabrizi, Gert Wurzinger, Eva Traunmüller-Wurm, Kristina Kutics, Markus Rauter, Fikreta Grabcanovic-Musija, Simona Müller, Norbert Kaufmann, Judith Löffler-Ragg, Arschang Valipour, Georg-Christian Funk

**Affiliations:** 1 Karl Landsteiner Institute for Lung Research and Pulmonary Oncology, Klinik Ottakring, Montleartstraße 37, 1160 Vienna, Austria; 2 Division of Gastroenterology and Hepatology, Department of Medicine III, Medical University of Vienna, Vienna, Austria; 3 Department of Medicine II With Pneumology, Klinik Ottakring, Vienna, Austria; 4 Center of Pulmology, LKH Graz II, Standort Enzenbach, Gratwein-Strassengel, Austria; 5 Department of Pulmology, Klinikum Wels-Grieskirchen, Wels, Austria; 6 Department of Pulmonology, Klinikum Klagenfurt Am Woerthersee, Klagenfurt, Austria; 7 Public Health Office, City of Salzburg, Salzburg, Austria; 8 Department of Pulmonology, Landeskrankenhaus Hohenems, Hohenems, Austria; 9 Division of Gastroenterology, Infectiology and Pneumology, Department of Medicine, LKH Graz II, Graz, Austria; 10 Department of Medicine II, Medical University of Innsbruck, Innsbruck, Austria; 11 Department of Respiratory and Critical Care Medicine, Klinik Floridsdorf, Vienna, Austria

**Keywords:** Alpha-1-antitrypsin, Alpha-1-antitrypsin deficiency, Diagnostic delay

## Abstract

**Background:**

Alpha-1-antitrypsin (AAT) deficiency (AATD) is a genetic disorder that can manifest as lung disease. A delay between onset of symptoms and diagnosis of AATD is common and associated with worse clinical status and more advanced disease stage but the influence on survival is unclear.

**Objective:**

We aimed to investigate the impact of diagnostic delay on overall survival (OS) and transplant-free survival (TS) in AATD patients.

**Methods:**

We analysed 268 AATD patients from the prospective multi-centre Austrian Alpha-1 Lung (AAL) Registry, employing descriptive statistics, Chi-square-test as well as univariable (Kaplan–Meier plots, log-rank test) and multivariable survival analysis (Cox regression).

**Results:**

The predominant phenotype was Pi*ZZ (82.1%). At diagnosis, 90.2% had an AAT level below 0.6 g/L. At inclusion, 28.2% had never smoked, 68.0% had quit smoking and 3.8% continued to smoke. Lung disease was diagnosed in 98.5%, thereof most patients were diagnosed with emphysema (63.8%) and/or chronic obstructive pulmonary disease (44.0%). Median diagnostic delay was 5.3 years (inter-quartile range [IQR] 2.2–11.5 years). In multivariable analysis (n = 229), a longer diagnostic delay was significantly associated with worse OS (hazard ratio [HR] 1.61; 95% CI 1.09–2.38; p = 0.016) and TS (HR 1.43; 95% CI 1.08–1.89; p = 0.011), independent from age, smoking status, body mass index (BMI), forced expiratory volume in one second (FEV_1_) and long-term oxygen treatment. Furthermore, BMI, age and active smoking were significantly associated with worse OS as well as BMI, active smoking and FEV_1_ were with worse TS.

**Conclusions:**

A delayed diagnosis was associated with significantly worse OS and TS. Screening should be improved and efforts to ensure early AATD diagnosis should be intensified.

**Supplementary Information:**

The online version contains supplementary material available at 10.1186/s12931-023-02338-0.

## Background

Alpha-1-antitrypsin (AAT) deficiency (AATD) is a genetic disorder that can manifest as lung or liver disease [1]. It is caused by mutations of the gene *SERPINA1*, encoding the proteinase inhibitor (Pi) AAT [1]. Most of the severe AATD cases result from the homozygous amino acid replacement Glu342Lys (called “Z allele”), the Pi*ZZ genotype, which can lead to both lung and liver manifestations and is typically characterized by a low AAT serum level [1–3]. It is estimated that over 100,000 persons in Europe exhibit the Pi*ZZ genotype [4, 5].

An even more frequent variant is the “S allele” (amino acid substitution Glu264Val) which can, in heterozygous combination with the Z allele (Pi*SZ), cause hepatic fibrosis as well as increase the risk for COPD and emphysema in smokers [6–8]. Recent evidence suggests that persons bearing the heterozygous combination of Z allele and the wild-type M allele (Pi*MZ) might also have an increased risk to develop COPD or liver cirrhosis, particularly in combination with other risk factors, although the association is reported to be weaker than in Pi*SZ persons [9–13].

Pulmonary manifestations of AATD include COPD and emphysema, caused by chronic inflammation due to unopposed proteinase activity in the lungs [1, 14]. The insufficient quantity of (functional) AAT is further decreased by smoking, as oxidants in cigarette smoke oxidize the active-site methionines of AAT and increase its polymerization [15]. Indeed, many Pi*ZZ patients who smoke develop COPD and/or emphysema at an unusually young age and the risk for these conditions is significantly increased in persons with Pi*SZ and Pi*MZ phenotype who smoke [7, 8, 11].

Previous publications have demonstrated that a wide-spread problem in AATD is late diagnosis, often several years after the onset of first respiratory tract symptoms [16, 17]. Although it has been suggested that an early AATD diagnosis could be beneficial as it allows timely smoking cessation, family screening and initiation of treatment by AAT augmentation therapy [18], direct evidence of the harmfulness of a delayed AATD diagnosis is sparse. In a recent study, a longer diagnostic delay was associated with worse clinical status and more advanced disease stage [19]. However, to our best knowledge, no study about the impact of diagnostic delay in AATD patients on survival has yet been published.

Here, we report results from a large national multi-centre register, the Austrian Alpha-1 Lung (AAL) Registry. In particular, we investigated the impact of diagnostic delay and other factors on overall survival (OS), transplant-free survival (TS) and clinical characteristics.

## Methods

### Patients

The Austrian Alpha-1 Lung (AAL) Registry is a national multi-centre registry. All adult patients (≥ 18 years old) who were diagnosed by phenotyping or genotyping with AATD (any phenotype except Pi*MM) in one of nine specialised Austrian AATD centres (in alphabetical order: Klinik Ottakring; Klinikum Klagenfurt am Woerthersee; Klinikum Wels-Grieskirchen; Landeskrankenhaus Hohenems; Landeskrankenhaus Natters; LKH Graz II—West; LKH Graz II—Enzenbach; Medical University of Innsbruck; Paracelsus Medical University Salzburg) could be entered into the registry, provided that the patient gave written, informed consent. Exclusion criteria were phenotype Pi*MM and refusal to give consent.

The AAL Registry was established in 2010 and patients were prospectively included from this year on. Furthermore, data of 145 Austrian patients from the retrospective Alpha1-international-registry (AIR), containing data of patients with the phenotypes Pi*ZZ, Pi*SZ, Pi*S0 and Pi*00, who were included in the years 2002 to 2010 was transferred to the AAL Registry [17].

Routinely, check-up visits of Austrian AATD patients are scheduled once per year in specialized expert centres and, if required, in closer intervals in the extramural setting. In Austria, augmentation therapy is mainly available at specialized AATD expert centres. The decision if a patient is offered augmentation therapy is based on local guidelines [20]. In general, augmentation therapy should be considered in non-smoking patients with severe AATD and impaired lung function (FEV_1_ ≤ 65%) or rapid decline of lung function. Augmentation therapy is generally not recommended for patients with FEV_1_ < 30% [20].

### Data collection

Data was entered into the AAL Registry by the treating physician via web-based electronic case report form (eCRF). At inclusion, demographic and biometrical data as well as clinical parameters were recorded. Forced expiratory volume in 1 s (FEV_1_) after application of bronchodilators was employed to assess lung function. The time of first onset of any respiratory tract-related symptoms such as coughing or dyspnoea that subjectively marks the beginning of symptomatic lung disease was recorded as (retrospectively) reported by the patient. If a patient had been diagnosed with manifest lung disease (e.g. COPD, emphysema, etc.), was recorded at inclusion into the registry as self-reported by the patient.

### Statistical analyses and definition of endpoints

In order to provide a clear picture of factors that are related to survival among the relevant population, all statistical analyses were restricted to the population of interest for this study, i.e. patients for whom data on survival and diagnostic delay were available.

Baseline patient characteristics are presented using descriptive statistics. Diagnostic delay was defined as the time from the first onset of respiratory tract-related symptoms such as coughing or dyspnoea until the day of AATD diagnosis. In order to analyse the association of diagnostic delay and other characteristics, differences between patients with shorter vs. longer diagnostic delay were evaluated by Chi-square-test.

Overall survival (OS) was defined as the time from AATD diagnosis until death, regardless of the cause of death. In order to assess a combined endpoint, transplant-free survival (TS) was defined as the time from AATD diagnosis until death or the date of receiving lung transplantation. Patients who were still alive on April 15, 2020, (end of follow-up) or who were lost to follow-up were censored at the time of last contact. Kaplan–Meier plots and log-rank test were employed for univariable analysis of survival. For univariable OS/TS analysis of diagnostic delay, a cut-off of 2 years was chosen, in order to compare the groups of patients with shorter (< 2 years) vs. longer (> 2 years) diagnostic delay, as we assumed that this should be the absolute maximum what would be a clinically acceptable time for AATD diagnosis. Median follow-up was calculated using the reverse Kaplan–Meier method.

Multivariable survival analysis was done by Cox regression. The proportionality assumption was assessed using time interaction variables, separately for every independent variable. Apart from diagnostic delay which is the main variable of interest of our study, we included well-known predictors of outcome in AATD/COPD patients, namely age, smoking status, FEV_1_, BMI and long-term oxygen treatment, based on biological plausibility and previous literature. We did not include variables that are known confounders of FEV_1_ (or other variables in the multivariable model), e.g. diagnosis of emphysema and use of antiobstructive therapy, in order to avoid collinearity in the multivariable model. For multivariable survival analysis as a continuous variable, the variable “diagnostic delay” was transformed (logarithmic transformation using the natural logarithm), in order to achieve normal distribution.

A p-value < 0.05 was considered statistically significant. Wherever multiple testing was used, the level of statistical significance was corrected for multiple testing using the Bonferroni method. Wherever applicable, the level of statistical significance calculated by Bonferroni method is given in the respective table. Statistical analysis was performed using IBM SPSS version 27.0 (Amonk, NY, USA).

### Ethical considerations

The study was approved by the human research ethics committee of the City of Vienna on January 31, 2012 (number EK11-248-0112). The study protocol conforms to the ethical guidelines of the Declaration of Helsinki and the Good Scientific Practice guidelines of the Medical University of Vienna. Written, informed consent was obtained from all patients who were included in the study.

## Results

### Patient characteristics

**Fig. 1:**
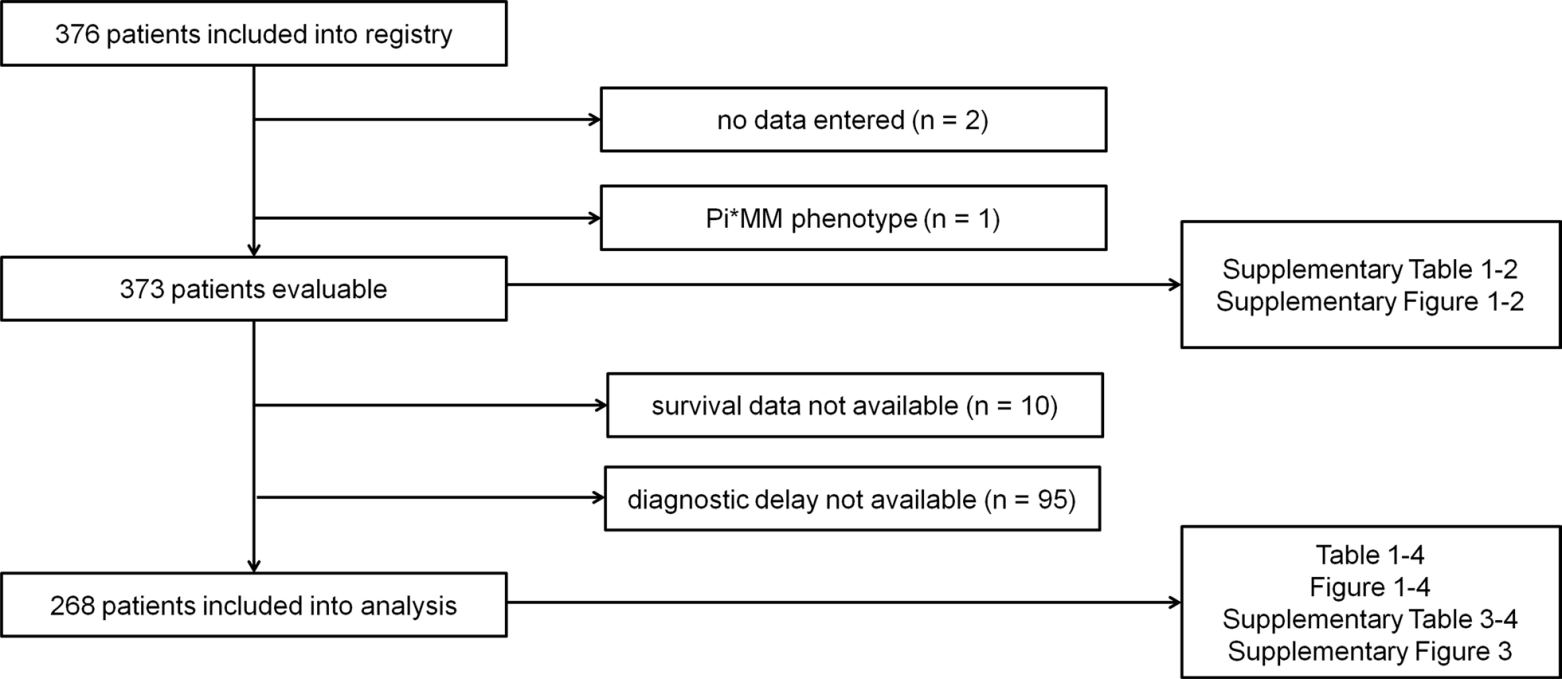
Patient flow chart

In total, 376 patients were included in the database. Two patients whose datasets did not contain any data and one patient with Pi*MM phenotype and no documented AAT serum level below 1.1 g/L were a priori excluded from analysis. Thus, 373 patients were evaluable (patient flow chart, Fig. 1). Median time from AATD diagnosis to inclusion into registry was 8.8 months (inter-quartile range [IQR] 1.4–76.9 months). Baseline characteristics of the whole study cohort (373 patients) are presented in Additional file 1: Table S1, more detailed information on their phenotypes in Additional file 2: Table S2. Boxplots of serum AAT level and forced expiratory volume in 1 s (FEV_1_) according to phenotype are shown in Additional file 5: Fig. S1, Additional file 6: Fig. S2, respectively.

**Fig. 2:**
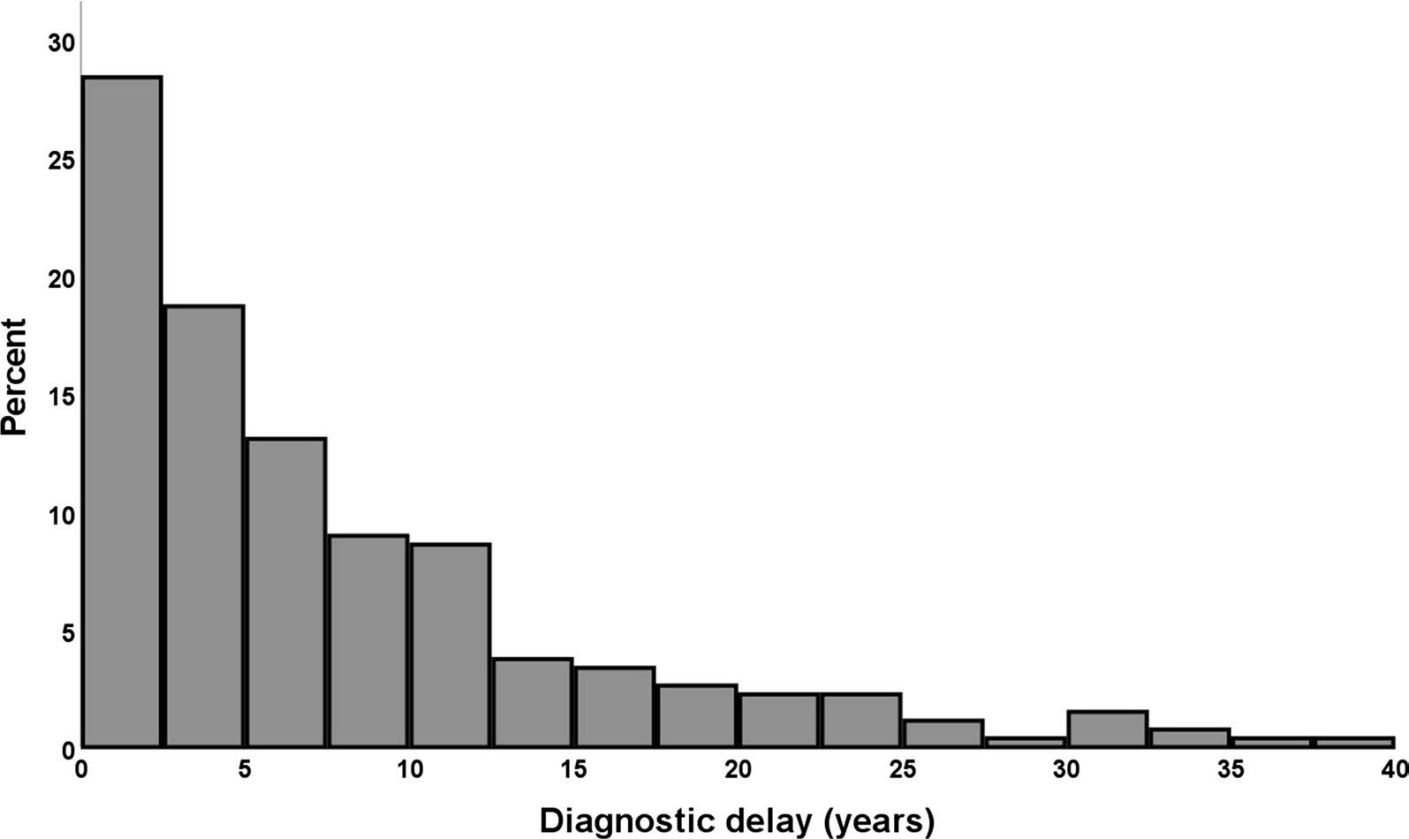
Diagnostic delay

All further analyses were conducted in the population of interest, i.e. the 268 patients for whom data on diagnostic delay, defined as the time from the first onset of respiratory tract-related symptoms until AATD diagnosis, and survival were available (patient flow chart, Fig. 1). Median diagnostic delay was 5.3 years (inter-quartile range [IQR] 2.2–11.5 years) (Fig. 2).

The baseline characteristics of the population of interest (n = 268) are presented in Table 1. The majority of patients exhibited a Pi*ZZ phenotype (n = 220; 82.1%). At inclusion into the registry, 75 patients (28.2%) had never smoked, 181 patients (68.0%) had quit smoking and 10 patients (3.8%) continued to smoke. At diagnosis, 90.2% of the patients had an AAT level below 0.6 g/L which is the threshold above which individuals are assumed to be protected from severe lung manifestations [8, 21]. Lung disease was diagnosed in 264 patients (98.5%), thereof most patients were diagnosed with emphysema (63.8%) and/or chronic obstructive pulmonary disease (44.0%).

**Table 1:** Baseline patient characteristics of the population of interest (n = 268)

Variable		n	%
Total		268	100
Sex (n = 268)	Male	166	61.9
	Female	102	38.1
Age (years) (n = 268)	Mean (standard deviation)	53.4 (11.9)	
Diagnostic delay (years) (n = 268)	Median (IQR)	5.3 (2.2–11.5)	
	< 2	62	23.1
	2–5	66	24.6
	5–10	57	21.3
	> 10	83	31.0
Phenotype (n = 268)	ZZ	220	82.1
	SZ	29	10.8
	Other	19	7.1
Serum AAT level (g/L) at time of AATD diagnosis (n = 163)	Mean (standard deviation)	0.33 (0.19)	
	≥ 0.6	16	9.8
	< 0.6	147	90.2
Body mass index (BMI; kg/m^2^) (n = 268)	Mean (standard deviation)	24.2 (4.0)	
Smoking status (n = 266)	Never	75	28.2
	Yes, at any time	191	71.8/100
	Yes, ex-smoker	181	68.0/94.8
	Yes, active smoking	10	3.8/5.2
Reason for being tested (n = 263)	Symptomatic disease	243	92.4
	Family-based screening	20	7.6
Lung disease, as self-reported by the patient (multiple diagnoses possible) (n = 268)	None	4	1.5
	Any lung disease	264	98.5
	Chronic obstructive pulmonary disease (COPD)	118	44.0
	Emphysema	171	63.8
	Chronic bronchitis	74	27.6
	Asthma	29	10.8
	Bronchiectasis	13	4.9
	Lung cancer	0	0.0
Respiratory tract-related symptoms (multiple symptoms possible) (n = 268)	None	0	0.0
	Any respiratory tract-related symptoms	268	100
	Cough	53	19.8
	Dyspnea	236	88.1
Forced expiratory volume in 1 s (FEV_1_) in % of the expected value (n = 265)	Mean (standard deviation)	52.5 (24.7)	
	≤ 50%	143	54.0
	> 50%	122	45.5
Cardiovascular comorbidity (n = 268)	No	244	91.0
	Yes	24	9.0
History of pneumonia (n = 261)	No	162	62.1
	Yes	99	37.9
History of exacerbation (n = 157)	No	101	64.3
	Yes	56	35.7
History of lung transplantation (n = 268)	No	263	98.1
	Yes	5	1.9
History of lung volume reduction surgery (n = 268)	No	263	98.1
	Yes	5	1.9
Treatment with inhalative antiobstructive agents (n = 268)	No	25	9.3
	Yes	243	90.7
Long-term oxygen therapy (n = 266)	No	216	81.2
	Yes	50	18.8
AAT augmentation therapy (n = 268)	No	157	58.6
	Yes	111	41.4

If not stated otherwise, values at time of inclusion into registry

*AAT* alpha-1-antitrypsin, *AATD* alpha-1-antitrypsin deficiency, *BMI* body mass index, *COPD* chronic obstructive pulmonary disease, *FEV*_*1*_ forced expiratory volume in 1 s, *IQR* inter-quartile range

### Analysis of overall survival

Of 268 patients analysed, 24 patients (9.0%) died during follow-up. Median follow-up was 85.2 months. In the whole cohort, survival rates were 96.2% after 5 years, 92.0% after 10 years and 85.5% after 15 years.

**Fig. 3:**
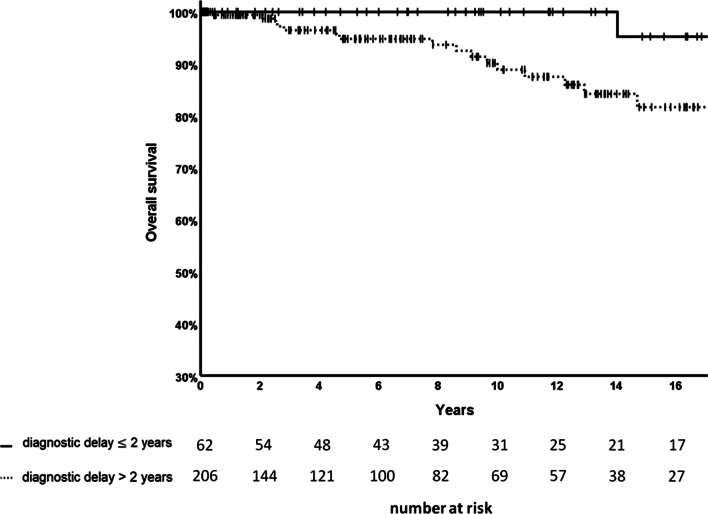
Kaplan–Meier plot of overall survival (OS) by diagnostic delay ≤/> 2 years (n = 268)

In univariable analysis, patients with a long diagnostic delay (> 2 years) had a lower OS (15-year survival rate 81.7% vs. 95.2%) than patients with a shorter diagnostic delay (≤ 2 years) (log-rank p-value = 0.080; Fig. 3). Active smoking at inclusion into registry (yes vs. no: 10-year survival rate 0.0% vs. 93.2%) was significantly associated with dismal survival (log-rank p-value < 0.001). Yet, the survival of ex-smokers and never-smokers was similar (15-year survival rate 86.5% vs. 86.5%) (log-rank p-value = 0.916; Additional file 7: Figure S3). OS was also significantly lower in patients with low FEV_1_ and long-term oxygen treatment (Table 2).

**Table 2:** Univariable analysis of overall survival (OS)

Variable		n	5-year survival rate, %	10-year survival rate, %	15-year survival rate, %	p value (log-rank test)
Total		268	96.2	92.0	85.5	–
Sex (n = 268)	Male	166	96.1	90.0	83.3	0.745
	Female	102	96.5	96.5	89.6	
Age at inclusion into registry (years) (n = 268)	≤ 65	224	96.8	92.8	86.6	0.042
	> 65	44	93.4	87.9	80.6	
Diagnostic delay (years) (n = 268)	≤ 2	62	100	100	95.2	0.080
	> 2	206	94.8	88.9	81.7	
Phenotype (n = 268)	Pi*ZZ	220	96.9	92.4	85.6	0.633
	other	48	91.7	91.7	91.7	
Body mass index (BMI) at inclusion into registry (kg/m^2^) (n = 268)	≤ 25	168	95.1	91.2	82.9	0.071
	> 25	100	98.7	93.8	90.4	
Smoking status at time of inclusion into registry (n = 266)	Never	75	98.5	95.9	86.5	0.002
	Yes, ex-smoker	181	95.8	92.0	86.5	
	Yes, active smoking	10	80.0	0.0	0.0	
Active smoking at time of inclusion into registry (n = 266)	No	256	96.7	93.2	86.6	< 0.001
	Yes	10	80.0	0.0	0.0	
Reason for being tested (n = 263)	Symptomatic disease	243	96.4	92.5	84.9	0.324
	Family-based screening	20	94.1	85.6	85.6	
Diagnosis of chronic obstructive pulmonary disease (COPD) at inclusion into registry, as self-reported by the patient (n = 268)	No	150	96.7	93.4	88.8	0.101
	Yes	118	95.4	88.1	70.2	
Diagnosis of emphysema at inclusion into registry, as self-reported by the patient (n = 268)	No	97	100	95.7	95.7	0.092
	Yes	171	94.7	90.5	82.6	
Forced expiratory volume in 1 s (FEV_1_) in % of the expected value at inclusion into registry (n = 265)	≤ 50%	143	94.5	89.3	80.2	0.004
	> 50%	122	99.0	96.7	96.7	
Cardiovascular comorbidity (n = 268)	No	244	96.5	92.8	85.5	0.180
	Yes	24	91.7	83.3	83.3	
History of exacerbation at inclusion into registry (n = 157)	No	101	98.8	95.0	95.0	0.011
	Yes	56	89.6	83.6	54.9	
Treatment with inhalative antiobstructive agents at inclusion into registry (n = 268)	No	25	100	88.9	88.9	0.561
	Yes	243	95.8	92.2	85.1	
Long-term oxygen therapy at inclusion into registry (n = 266)	No	216	98.0	93.5	90.6	0.002
	Yes	50	88.2	84.6	68.5	
AAT augmentation therapy at inclusion into registry (n = 268)	No	157	92.4	88.0	85.4	0.308
	Yes	111	100	95.8	86.8	

Statistical significance (corrected by Bonferroni method): p value < 0.0031

*AAT* alpha-1-antitrypsin, *AATD* alpha-1-antitrypsin deficiency, *BMI* body mass index, *COPD* chronic obstructive pulmonary disease, *FEV*_*1*_ forced expiratory volume in 1 s

Results of multivariable analysis of OS by Cox regression (n = 229) are presented in Table 3. A longer diagnostic delay was significantly associated with worse survival (hazard ratio [HR] 1.61; 95% CI 1.09–2.38; p = 0.016), independent from age, active smoking, BMI, pulmonary function (FEV_1_) and long-term oxygen treatment.

**Table 3:** Multivariable analysis of overall survival (OS) by Cox regression (n = 229)

Variable		HR	95% CI	p value (Cox regression)
Diagnostic delay (years)*—continuous		1.61	1.09–2.38	0.016
Body mass index (BMI; kg/m^2^)—continuous		0.86	0.73–1.00	0.049
Age (years)—continuous		1.07	1.02–1.12	0.009
Active smoking at time of inclusion into registry	No	1.00	–	0.006
	Yes	11.54	2.02–65.83	
Forced expiratory volume in 1 s (FEV_1_) in % of the expected value	> 50	1.00	–	0.141
	≤ 50	3.23	0.68–15.36	
Long-term oxygen therapy at inclusion into registry	No	1.00	–	0.107
	Yes	2.33	0.83–6.50	

*After logarithmic transformation (using natural logarithm)

*BMI* body mass index, *CI* confidence interval, *FEV*_*1*_ forced expiratory volume in 1 s, *HR* hazard ratio

### Analysis of transplant-free survival

**Fig. 4:**
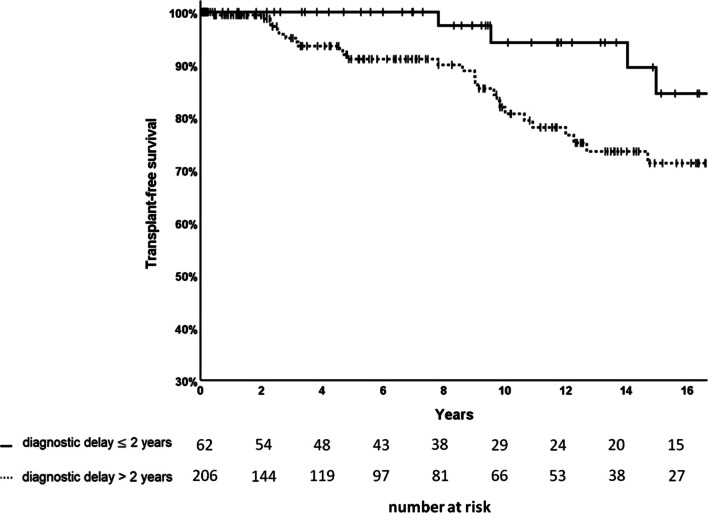
Kaplan–Meier plot of transplant-free survival (TS) by diagnostic delay ≤/> 2 years (n = 268)

Of 268 analysed patients, 24 (9.0%) died during follow-up and five (1.9%) received a lung transplant. A diagnostic delay of more than 2 years was associated with a lower transplant-free survival (TS) (15-year TS rate 71.3% vs. 84.5%), compared to a diagnostic delay ≤ 2 years; (log-rank p-value = 0.065; Fig. 4). Active smoking at inclusion into the registry (10-year TS rate 0.0% vs. 85.1%) was significantly associated with poor TS (log-rank p-value < 0.001). Further results of univariable TS analysis are presented in the Additional file 3: Table S3. In multivariable analysis by Cox regression (n = 229), longer diagnostic delay was associated with a significantly lower TS (HR 1.43; 95% CI 1.08—1.89; p = 0.011), independent from age, active smoking, BMI and pulmonary function (FEV_1_) (Additional file 4: Table S4).

### Association of diagnostic delay with other variables

Results of the comparison of the characteristics of patients according to diagnostic delay (≤ 2 vs. > 2 years) are presented in Table 4. There was no significant association of diagnostic delay (≤ 2 vs. > 2 years) with any of the analysed patient characteristics.

**Table 4:** Association of diagnostic delay with other variables

Variable		n	diagnostic delay (years)				p value (Chi-square)
			≤ 2		> 2		
			n	%	n	%	
Total		268	62	100%	206	100%	–
Sex	Male	166	37	59.7%	129	62.6%	0.676
Age at inclusion into registry (years)	> 65	44	7	11.3%	37	18.0%	0.214
Phenotype	ZZ	220	55	88.7%	165	80.1%	0.121
Serum AAT level (g/L) at time of AATD diagnosis*	≤ 0.6	147	32	94.1%	115	89.1%	0.386
Forced expiratory volume in 1 s (FEV_1_) at inclusion into registry in % of the expected value^†^	≤ 50	143	31	50.0%	112	55.2%	0.475
Active smoking at inclusion into registry°		10	2	3.2%	8	3.9%	0.801
Being tested because of symptomatic disease^$^		243	55	88.7%	188	93.1%	0.453
Cardiovascular comorbidity		24	6	9.7%	18	8.7%	0.820
History of exacerbation at inclusion into registry^x^		56	10	28.6%	46	37.7%	0.320
Long-term oxygen therapy at inclusion into registry°		50	9	14.8%	41	20.0%	0.357
AAT augmentation therapy at inclusion into registry		111	30	48.4%	81	39.3%	0.204

*Missing values: n = 105

^†^Missing values: n = 3

°Missing values: n = 2

^$^Missing valus: n = 5

^x^Missing values: n = 111

Statistical significance (corrected by Bonferroni method): p value < 0.0045

*AAT* Alpha-1-antitrypsin, *AATD* alpha-1-antitrypsin deficiency, *BMI* body mass index, *COPD* chronic obstructive pulmonary disease, *FEV*_*1*_ forced expiratory volume in 1 s

With 10 years as a cut-off for diagnostic delay, in the group with longer delay (> 10 years), there was a significantly higher proportion of patients with a history of pneumonia (42.4% vs. 24.1%, p = 0.002) and exacerbation (50.0% vs. 28.7%, p = 0.008) at inclusion into the registry.

## Discussion

In this study, we investigated the impact of diagnostic delay on overall survival (OS) and transplant-free survival (TS) in a prospective multi-centre cohort of 268 alpha-1-antitrypsin deficiency (AATD) patients from the Austrian Alpha-1 Lung (AAL) Registry. We observed that a longer diagnostic delay was associated with significantly worse overall survival and transplant-free survival, independent from known risk factors such as age, BMI, impaired pulmonary function, smoking status and long-term oxygen treatment. These findings suggest that screening in relevant patient groups (e.g. COPD patients) has to be improved and intensified efforts to ensure early AATD diagnosis are necessary.

Diagnostic delay is a wide-spread problem in AATD and the diagnosis is often made several years after the onset of first respiratory tract symptoms [1, 19]. A delayed AATD diagnosis has been shown to be associated with worse clinical and functional status, worsened air-flow obstruction, and negative psychosocial consequences [19, 22]. Yet, to our best knowledge, no evidence was existing previously to our study to elucidate whether a delayed AATD diagnosis is associated with survival. Our findings suggest that a diagnostic delay does not only translate to higher disease severity but also leads to higher mortality.

The fact that there was a significantly higher percentage of patients with a history of pneumonia and exacerbation in the group with a diagnostic delay above 10 years, might indicate a cause of their diminished survival: Exacerbations are well known to predict poor outcome in COPD as well as AATD [23, 24]. A possible reason for the association of diagnostic delay and exacerbations might be that in patients with timely diagnosis interventions such as smoking cessation can be undertaken to decelerate disease progression and to reduce the risk of exacerbations. Augmentation therapy that can only be initiated after AATD diagnosis might also play a role although this remains unclear at the moment. Particularly, the hypothesis that augmentation treatment might reduce the frequency of exacerbation is currently under investigation, however with no final clarity yet [1, 20].

In 2003, the joint ATS/ERS statement recommended AATD testing in patients with emphysema, COPD, asthma with incompletely reversible obstruction, unexplained liver disease, bronchiectasis without evident etiology as well as in relatives of AATD patients [25]. Later, the Alpha-1 Foundation Clinical Practice Guidelines recommend testing in all patients with COPD, unexplained chronic liver disease, unexplained bronchiectasis and in relatives of AATD patients [26]. In contrast, the 2017 European Respiratory Society (ERS) statement recommends testing in all patients with COPD or adult-onset asthma but not in bronchiecatsis patients [24].

Adherence to these recommendations seems to be incomplete: A recent study found that in a cohort of COPD patients only 2% were tested although AATD prevalence was 24% in the tested patients [27]. Another recent analysis revealed that 9% of COPD/emphysema and 7% of bronchiectasis patients in a large Central and Eastern European cohort were bearing the Pi*ZZ phenotype, confirming that the detection of AATD in these populations is necessary [28]. Yet, the question if brochiectasis patients should be tested for AATD remains unclear as a recent study from the United Kingdom observed that only 0.5% of 1,600 tested bronchiectasis patients had severe AATD [29].

Continuation to smoke was a predictor of unfavourable overall survival in our cohort, both in uni- and multivariable analysis. These findings—which are in line with many previous studies [1, 15]—highlight the paramount importance of smoking cessation, at the latest when the diagnosis of AATD has been made. In particular, smoking is associated with a high risk of developing lung disease and an accelerated decline of lung function in AATD patients [1, 15, 18]. Hence, patients with AATD should be strongly advised to quit smoking, regardless of their clinical presentation.

Most patients in our cohort were tested for AATD because they were diagnosed with lung disease and referred for treatment to a specialised centre. Only a minority was screened because of relatives with AATD. For this reason, our registry might not be representative for all persons in Austria bearing an AATD phenotype. However, it has to be noted that the primary focus on symptomatic AATD patients is a key strength of our registry: Many of the patients in our cohort even suffer from severely impaired lung function and information on such severely affected patients is crucial for improving their outcome. Thus, we are confident that our findings add important information to the literature and extend the current body of knowledge.

The onset of respiratory tract-related symptoms that subjectively marks the beginning of symptomatic lung disease was recorded as (retrospectively) reported by the patient. This might be a limitation of this study but the diagnostic delay and the reported symptoms in our cohort were in a similar range as results from previous studies [16, 19, 22]. Hence, it can be assumed that onset of first symptoms and diagnostic delay of our cohort are representative. Another limitation might be that most variables were not obtained at the time of AATD diagnosis or the first onset of symptoms but at inclusion into the registry. Yet, as the time between diagnosis and inclusion into the registry was short (median 8.8 months), it can be assumed that, in most patients, the values obtained at inclusion into the registry are similar to those at the time of AATD diagnosis.

It is assumed that AATD is an underdiagnosed disease and population-wide screening programmes would probably reveal many asymptomatic or pre-symptomatic carriers of typical AATD phenotypes with an expected ratio of one in 3,500 screened persons [4, 5, 30, 31]. However, recent evidence suggests that even many AATD patients with symptomatic lung disease are not correctly identified: Only 6% of the Pi*ZZ carriers in a large biobank were diagnosed although they had an eightfold increased risk of COPD, a sevenfold increased risk of emphysema and 2.4-fold increased risk of mortality, compared to wild-type (Pi*MM) [32]. One reason might be the low awareness of physicians and consequently low adherence to the guidelines [30, 33]. Except earlier onset in some cases, the clinical presentation of COPD patients with or without AATD is similar which might be another obstacle for timely diagnosis [31, 33].

These obstacles have to be overcome since early AATD diagnosis is crucial for improving outcome: As our study and many others reported smoking as an essential risk factor for unfavourable outcome in AATD patients, an immediate diagnosis makes timely smoking cessation possible. For this reason, it is even beneficial to identify asymptomatic persons with AATD phenotypes. In many of them, the development of lung disease might be prevented or at least the progression might be slowed down by smoking cessation, as previous studies have suggested [18, 32]. A Swedish study found a reduced survival time in PiZZ subjects compared to randomly selected controls but the never-smoking PiZZ individuals had a similar life expectancy to the never-smokers in the general population, suggesting that finding AATD patients as early as possible and, thus, enabling timely smoking cessation is crucial for improving their outcome [34].

Furthermore, patients who are diagnosed immediately after developing symptoms could profit from earlier initiation of intravenous augmentation therapy which decelerates the loss of lung density and is associated with improved survival [35–38]. Recruiting of study participants for clinical trials in AATD is challenging due to a low number of identified AATD patients, even symptomatic ones [1, 31]. Intensified screening efforts would raise the number of potential participants and, thus, help researchers to improve feasibility and significance of clinical trials in AATD [31]. In addition, early AATD diagnosis is useful for timely screening of family members who might profit from pre-symptomatic AATD diagnosis and lifestyle modification [17, 31].

In order to achieve early AATD diagnosis and identification of carriers, the (re-)introduction of neonatal screening has been suggested but feasibility is difficult and costs are high whereas the ratio of identified carriers per screened person would be expected to be low [18, 31]. Hence, more targeted approaches are discussed since the number of correctly diagnosed AATD carriers is low even in patients with symptomatic lung disease [27, 31]. Sequencing of the *SERPINA1* gene in COPD patients was recently proposed as a possible tool for testing large cohorts [39]. Other ideas to improve AATD detection rates include family-based testing, detection of potential AATD patients by analysis of routinely collected electronic medical record (EMR) data, algorithm-based reminders within EMR software, and direct testing for AATD in pulmonary function testing laboratories [31, 40]. As a recent study reported a profound lack of knowledge about rare respiratory diseases among paediatricians and medical students, increased training of (future) medical professionals by adding specific subjects to the medical curriculum might also improve the proportion of timely diagnosed AATD [41].

## Conclusions

In conclusion, a delayed diagnosis of alpha-1-antitrypsin deficiency was associated with significantly worse overall survival and transplant-free survival in our cohort, independent from age, smoking status, BMI, pulmonary function and long-term oxygen treatment. This suggests that efforts to ensure early AATD diagnosis should be intensified. Particularly, in accordance with the relevant guidelines, all patients with COPD, emphysema, poorly responsive or late-onset asthma, and relatives of AATD patients should be tested.

## Supplementary Information

**Additional file 1: Table S1.** Baseline patient characteristics of the total population of the registry (n = 373).

**Additional file 2: Table S2.** Phenotypes of the total population of the registry.

**Additional file 3: Table S3.** Univariable analysis of transplant-free survival (TS) of the population of interest (n = 268).

**Additional file 4: Table S4.** Multivariable analysis of transplant-free survival (TS) by Cox regression (n = 229).

**Additional file 5: Figure S1.** Boxplot of serum alpha-1-antitrypsin (AAT) level at diagnosis for different phenotypes: Pi*ZZ patients had a significantly lower serum AAT level at diagnosis (mean ± SD: 0.253 ± 0.090 g/L) than Pi*SZ patients (0.584 ± 0.116 g/L; p < 0.001) or patients with other phenotypes (0.532 ± 0.292 g/L; p < 0.001).

**Additional file 6: Figure S2.** Boxplot of forced expiratory volume in 1 s (FEV_1_) in % at inclusion into registry for different phenotypes: FEV_1_ in % of the expected value at last measurement before inclusion into registry was also significantly lower in Pi*ZZ patients (mean ± SD: 61.4 ± 31.0%) than in Pi*SZ patients (77.8 ± 31.5%; p = 0.003) or patients with other phenotypes (76.1 ± 32.8%; p = 0.007).

**Additional file 7: Figure S3.** Kaplan–Meier plot of overall survival (OS) by smoking status at inclusion into registry, never-smokers vs. ex-smokers vs. current smokers (n = 266).

## Data Availability

The datasets generated and analysed during the current study are not publicly available but are available from the corresponding author on reasonable request.

## Abbreviations

AAL Registry: Austrian Alpha-1 Lung Registry
AAT: Alpha-1-antitrypsin
AATD: Alpha-1-antitrypsin deficiency
AIR: Alpha1-international-registry
BMI: Body mass index
CI: Confidence interval
COPD: Chronic obstructive pulmonary disease
CT: Computed tomography
eCRF: Electronic case report form
EMR: Electronic medical record
ERS: European Respiratory Society
FEV_1_: Forced expiratory volume in one second
HR: Hazard ratio
IQR: Inter-quartile range
OS: Overall survival
Pi: Proteinase inhibitor
SD: Standard deviation
TS: Transplant-free survival

## References

[1] Strnad P, McElvaney NG, Lomas DA (2020). Alpha 1 -antitrypsin deficiency. N Engl J Med.

[2] Jeppsson J-O (1976). Amino acid substitution Glu→Lys in α 1 -antitrypsin PiZ. FEBS Lett.

[3] Hamesch K, Mandorfer M, Pereira VM (2019). Liver fibrosis and metabolic alterations in adults with alpha-1-antitrypsin deficiency caused by the Pi*ZZ mutation. Gastroenterology.

[4] Blanco I, Bueno P, Diego I (2017). Alpha-1 antitrypsin Pi*Z gene frequency and Pi*ZZ genotype numbers worldwide: an update. Int J Chron Obstruct Pulmon Dis.

[5] Greulich T, Nell C, Hohmann D (2017). The prevalence of diagnosed α 1 -antitrypsin deficiency and its comorbidities: results from a large population-based database. Eur Respir J.

[6] Mandorfer M, Bucsics T, Hutya V (2018). Liver disease in adults with α1-antitrypsin deficiency. United Eur Gastroenterol J.

[7] McElvaney GN, Sandhaus RA, Miravitlles M (2020). Clinical considerations in individuals with α 1 -antitrypsin PI*SZ genotype. Eur Respir J.

[8] Franciosi AN, Hobbs BD, McElvaney OJ (2020). Clarifying the risk of lung disease in SZ alpha-1 antitrypsin deficiency. Am J Respir Crit Care Med.

[9] Schaefer B, Mandorfer M, Viveiros A (2018). Heterozygosity for the alpha-1-antitrypsin Z allele in cirrhosis is associated with more advanced disease. Liver Transplant.

[10] Strnad P, Buch S, Hamesch K (2019). Heterozygous carriage of the alpha1-antitrypsin Pi*Z variant increases the risk to develop liver cirrhosis. Gut.

[11] Molloy K, Hersh CP, Morris VB (2014). Clarification of the risk of chronic obstructive pulmonary disease in α 1 -antitrypsin deficiency PiMZ heterozygotes. Am J Respir Crit Care Med.

[12] Foreman MG, Wilson C, DeMeo DL (2017). Alpha-1 Antitrypsin PiMZ genotype is associated with chronic obstructive pulmonary disease in two racial groups. Ann Am Thorac Soc.

[13] Ferkingstad E, Oddsson A, Gretarsdottir S (2018). Genome-wide association meta-analysis yields 20 loci associated with gallstone disease. Nat Commun.

[14] Guyot N, Wartelle J, Malleret L (2014). Unopposed Cathepsin G, neutrophil elastase, and proteinase 3 cause severe lung damage and emphysema. Am J Pathol.

[15] Alam S, Li Z, Janciauskiene S (2011). Oxidation of Z α 1 -antitrypsin by cigarette smoke induces polymerization. Am J Respir Cell Mol Biol.

[16] Stoller JK, Sandhaus RA, Turino G (2005). Delay in diagnosis of α1-antitrypsin deficiency. Chest.

[17] Huber F, Schmid-Scherzer K, Wantke F (2010). Alpha1-Antitrypsin-Mangel in Österreich: Auswertung der österreichischen Datenbank des internationalen Alpha1-Antitrypsin Registers. Wien Klin Wochenschr.

[18] Wall M, Moe E, Eisenberg J (1990). Long-term follow-up of a cohort of children with alpha-1-antitrypsin deficiency. J Pediatr.

[19] Tejwani V, Nowacki AS, Fye E (2019). The impact of delayed diagnosis of alpha-1 antitrypsin deficiency: the association between diagnostic delay and worsened clinical status. Respir Care.

[20] Greulich T, Fähndrich S, Clarenbach C (2020). Alpha-1-Antitrypsin-Mangel (AATM)—Ein Expertenstatement. Pneumologie.

[21] Brantly ML, Wittes JT, Vogelmeier CF (1991). Use of a highly purified α1-antitrypsin standard to establish ranges for the common normal and deficient α1-antitrypsin phenotypes. Chest.

[22] Stoller JK, Smith P, Yang P (1994). Physical and social impact of alpha 1-antitrypsin deficiency: results of a survey. Cleve Clin J Med.

[23] Soler-Cataluña JJ, Martínez-García MA, Román Sánchez P (2005). Severe acute exacerbations and mortality in patients with chronic obstructive pulmonary disease. Thorax.

[24] Miravitlles M, Dirksen A, Ferrarotti I (2017). European Respiratory Society statement: diagnosis and treatment of pulmonary disease in α 1 -antitrypsin deficiency. Eur Respir J.

[25] American Thoracic Society, European Respiratory Society (2003). American Thoracic Society/European Respiratory Society statement: standards for the diagnosis and management of individuals with alpha-1 antitrypsin deficiency. Am J Respir Crit Care Med.

[26] Sandhaus RA, Turino G, Brantly ML (2016). The diagnosis and management of alpha-1 antitrypsin deficiency in the adult. Chronic Obstr Pulm Dis J COPD Found.

[27] Soriano JB, Lucas SJ, Jones R (2018). Trends of testing for and diagnosis of α 1 -antitrypsin deficiency in the UK: more testing is needed. Eur Respir J.

[28] Greulich T, Averyanov A, Borsa L (2017). European screening for alpha 1 -antitrypsin deficiency in subjects with lung disease. Clin Respir J.

[29] Carreto L, Morrison M, Donovan J (2020). Utility of routine screening for alpha-1 antitrypsin deficiency in patients with bronchiectasis. Thorax.

[30] Horváth I, Canotilho M, Chlumský J (2019). Diagnosis and management of α 1 -antitrypsin deficiency in Europe: an expert survey. ERJ Open Res.

[31] Brantly M, Campos M, Davis AM (2020). Detection of alpha-1 antitrypsin deficiency: the past, present and future. Orphanet J Rare Dis.

[32] Nakanishi T, Forgetta V, Handa T (2020). The undiagnosed disease burden associated with alpha-1 antitrypsin deficiency genotypes. Eur Respir J.

[33] Quinn M, Ellis P, Pye A (2020). Obstacles to early diagnosis and treatment of alpha-1 antitrypsin deficiency: current perspectives. Ther Clin Risk Manag.

[34] Tanash HA, Ekström M, Rönmark E (2017). Survival in individuals with severe alpha 1-antitrypsin deficiency (PiZZ) in comparison to a general population with known smoking habits. Eur Respir J.

[35] Dirksen A, Dijkman JH, Madsen F (1999). A randomized clinical trial of α1-antitrypsin augmentation therapy. Am J Respir Crit Care Med.

[36] Dirksen A, Piitulainen E, Parr DG (2009). Exploring the role of CT densitometry: a randomised study of augmentation therapy in 1-antitrypsin deficiency. Eur Respir J.

[37] Chapman KR, Burdon JGW, Piitulainen E (2015). Intravenous augmentation treatment and lung density in severe α1 antitrypsin deficiency (RAPID): a randomised, double-blind, placebo-controlled trial. Lancet.

[38] Rahaghi FF, Monk R, Ramakrishnan V (2020). Alpha-1 antitrypsin augmentation therapy improves survival in severely deficient patients with predicted FEV1 between 10% and 60%: a retrospective analysis of the NHLBI alpha-1 antitrypsin deficiency registry. Int J Chron Obstruct Pulmon Dis.

[39] Gupta N, Gaudreault N, Thériault S (2020). Granularity of SERPINA1 alleles by DNA sequencing in CanCOLD. Eur Respir J.

[40] Gurevich S, Daya A, Da Silva C (2021). Improving screening for alpha-1 antitrypsin deficiency with direct testing in the pulmonary function testing laboratory. Chronic Obstr Pulm Dis J COPD Found.

[41] Requena-Fernández MÁ, Dasí F, Castillo S (2020). Knowledge of rare respiratory diseases among paediatricians and medical school students. J Clin Med.

