# Supplementary material for: The impact of diagnostic delay on survival in alpha-1-antitrypsin deficiency: results from the Austrian Alpha-1 Lung Registry
Source: Respir Res. 2023 Jan 27;24:34. doi: 10.1186/s12931-023-02338-0 (PMC9881325; doi:10.1186/s12931-023-02338-0)
Supplement: Supplementary file 1 — Additional file 1: Table S1. Baseline patient characteristics of the total population of the registry (n = 373). [file 12931_2023_2338_MOESM1_ESM.docx]

| Supplementary Table 1. Baseline patient characteristics of the total population of the registry (n = 373). If not stated otherwise, values at time of inclusion into registry. | | | |
| --- | --- | --- | --- |
| Variable | | n | % |
| Total | | 373 | 100 |
| Sex (n = 373) | male | 213 | 57.1 |
|  | female | 160 | 42.9 |
| Age (years) (n = 373) | mean (standard deviation) | 50.0 (13.5) | |
| Diagnostic delay (years) (n = 272) | median (IQR) | 5.4 (2.2 - 11.5) | |
|  | < 2 | 62 | 22.8 |
|  | 2 - 5 | 68 | 25.0 |
|  | 5 - 10 | 58 | 21.3 |
|  | > 10 | 84 | 30.9 |
| Phenotype (n = 372) | ZZ | 293 | 78.8 |
|  | SZ | 42 | 11.3 |
|  | other | 37 | 9.9 |
| Serum AAT level (g/L) at time of AATD diagnosis (n = 215) | mean (standard deviation) | 0.35 (0.20) | |
|  | ≥ 0.6 | 34 | 15.8 |
|  | < 0.6 | 181 | 84.2 |
| Body mass index (BMI; kg/m²) (n = 372) | mean (standard deviation) | 24.2 (4.1) | |
| Smoking status (n = 369) | never | 126 | 34.1 |
|  | yes, at any time | 243 | 65.9/100 |
|  | yes, ex-smoker | 222 | 60.2/91.4 |
|  | yes, active smoking | 21 | 5.7/8.6 |
| In smokers: pack years (n = 361) | mean (standard deviation) | 21.5 (16.2) | |
| Reason for being tested (n = 355) | symptomatic disease | 283 | 79.7 |
|  | family-based screening | 72 | 20.3 |
| Lung disease, as self-reported by the patient (multiple diagnoses possible) (n = 373) | none | 92 | 24.7 |
|  | any lung disease | 281 | 75.3 |
|  | chronic obstructive pulmonary disease (COPD) | 123 | 33.0 |
|  | emphysema | 181 | 48.5 |
|  | chronic bronchitis | 81 | 21.7 |
|  | asthma | 30 | 8.0 |
|  | bronchiectasis | 14 | 3.8 |
|  | lung cancer | 0 | 0.0 |
| Respiratory tract-related symptoms (multiple symptoms possible) (n = 373) | none | 94 | 25.2 |
|  | any respiratory tract-related symptoms | 279 | 74.8 |
|  | cough | 56 | 15.0 |
|  | dyspnea | 246 | 66.0 |
| Forced expiratory volume in 1 second (FEV_1_) in % of the expected value (n = 369) | mean (standard deviation) | 64.9 (31.9) | |
|  | ≤ 50 % | 153 | 41.5 |
|  | > 50 % | 216 | 58.5 |
| Cardiovascular comorbidity (n = 373) | no | 346 | 92.8 |
|  | yes | 27 | 7.2 |
| History of pneumonia (n = 373) | no | 251 | 69.9 |
|  | yes | 108 | 30.1 |
| History of exacerbation (n = 219) | no | 162 | 74.0 |
|  | yes | 57 | 26.0 |
| History of lung transplantation (n = 373) | no | 367 | 98.4 |
|  | yes | 6 | 1.6 |
| History of lung volume reduction surgery (n = 373) | no | 367 | 98.4 |
|  | yes | 6 | 1.6 |
| Treatment with inhalative antiobstructive agents (n = 373) | no | 108 | 29.0 |
|  | yes | 265 | 71.0 |
| Long-term oxygen therapy (n = 371) | no | 316 | 85.2 |
|  | yes | 55 | 14.8 |
| AAT augmentation therapy (n = 373) | no | 252 | 67.6 |
|  | yes | 121 | 32.4 |
| Abbreviations: Alpha-1-Antitrypsin, AAT; Alpha-1-Antitrypsin Deficiency, AATD; body mass index, BMI; chronic obstructive pulmonary disease, COPD; forced expiratory volume in 1 second, FEV_1_; inter-quartile range, IQR; standard deviation, SD | | | |
