# Supplementary material for: The impact of diagnostic delay on survival in alpha-1-antitrypsin deficiency: results from the Austrian Alpha-1 Lung Registry
Source: Respir Res. 2023 Jan 27;24:34. doi: 10.1186/s12931-023-02338-0 (PMC9881325; doi:10.1186/s12931-023-02338-0)
Supplement: Supplementary file 2 — Additional file 2: Table S2. Phenotypes of the total population of the registry. [file 12931_2023_2338_MOESM2_ESM.docx]

| Supplementary table 2. Phenotypes of the total population of the registry. | | |
| --- | --- | --- |
| Phenotype | n | % |
| Total | 373 | 100 |
| Z/Z | 293 | 78.8 |
| S/Z | 42 | 11.3 |
| other | 37 | 9.9 |
| M/M-Malton | 6 | 1.6 |
| Z/I | 4 | 1.1 |
| Z/Q0-Clayton | 4 | 1.1 |
| Z/M-Malton | 3 | 0.8 |
| M/S | 3 | 0.8 |
| Z/M-Procida | 2 | 0.5 |
| S/S | 2 | 0.5 |
| M/Q0-Amersfoort | 2 | 0.5 |
| M/I | 2 | 0.5 |
| Z/M-Nichinan | 1 | 0.3 |
| Z-Augsburg/M-Malton | 1 | 0.3 |
| Z/M-Würzburg | 1 | 0.3 |
| Z/F | 1 | 0.3 |
| Z/new mutation (Arg63Gly) | 1 | 0.3 |
| Z/new mutation (not specified) | 1 | 0.3 |
| M/F | 1 | 0.3 |
| Q0-Amersfoort/Q0-Amerstfoort | 1 | 0.3 |
| VI/Q0-Amersfoort | 1 | 0.3 |
| missing values: n = 1 | | |
