# Supplementary material for: The impact of diagnostic delay on survival in alpha-1-antitrypsin deficiency: results from the Austrian Alpha-1 Lung Registry
Source: Respir Res. 2023 Jan 27;24:34. doi: 10.1186/s12931-023-02338-0 (PMC9881325; doi:10.1186/s12931-023-02338-0)
Supplement: Supplementary file 3 — Additional file 3: Table S3. Univariable analysis of transplant-free survival (TS) of the population of interest (n = 268). [file 12931_2023_2338_MOESM3_ESM.docx]

| Supplementary table 3. Univariable analysis of transplant-free survival (TS) of the population of interest (n = 268). | | | | | | |
| --- | --- | --- | --- | --- | --- | --- |
| Variable | | n | 5-year TS rate | 10-year TS rate | 15-year TS rate | p value (log rank test) |
| Total | | 268 | 93.5 % | 85.3 % | 74.7 % | - |
| Sex (n = 268) | male | 166 | 92.0 % | 86.2 % | 75.0 % | 0.726 |
|  | female | 102 | 96.5 % | 82.9 % | 73.0 % |  |
| Age at inclusion into registry (years) (n = 268) | ≤ 65 | 224 | 93.6 % | 84.9 % | 74.2 % | 0.601 |
|  | > 65 | 44 | 93.4 % | 87.9 % | 80.6 % |  |
| Diagnostic delay (years) (n = 268) | ≤ 2 | 62 | 100 % | 94.2 % | 84.5 % | 0.065 |
|  | > 2 | 206 | 91.1 % | 81.9 % | 71.3 % |  |
| Phenotype (n = 268) | Pi*ZZ | 220 | 94.5 % | 86.5 % | 75.4 % | 0.371 |
|  | other | 48 | 86.6 % | 74.2 % | 74.2 % |  |
| Body mass index (BMI) at inclusion into registry (kg/m²) (n = 268) | ≤ 25 | 168 | 91.0 % | 81.0 % | 69.3 % | 0.009 |
|  | > 25 | 100 | 98.7 % | 94.0 % | 85.2 % |  |
| Smoking status at time of inclusion into registry (n = 266) | never | 75 | 98.5 % | 90.7 % | 81.2 % | < 0.001 |
|  | yes, ex-smoker | 181 | 92.6 % | 85.1 % | 73.7 % |  |
|  | yes, active smoking | 10 | 60.0 % | 0.0 % | 0.0 % |  |
| Active smoking at time of inclusion into registry (n = 266) | no | 256 | 94.4 % | 86.8 % | 76.1 % | < 0.001 |
|  | yes | 10 | 60.0 % | 0.0 % | 0.0 % |  |
| Reason for being tested (n = 263) | symptomatic disease | 243 | 93.3 % | 84.9 % | 72.8 % | 0.996 |
|  | family-based screening | 20 | 94.1 % | 85.6 % | 85.6 % |  |
| Diagnosis of chronic obstructive pulmonary disease (COPD) at inclusion into registry, as self-reported by the patient (n = 268) | no | 150 | 95.9 % | 89.3 % | 79.4 % | 0.016 |
|  | yes | 118 | 89.0 % | 75.5 % | 58.1 % |  |
| Diagnosis of emphysema at inclusion into registry, as self-reported by the patient (n = 268) | no | 97 | 96.0 % | 91.8 % | 91.8 % | 0.053 |
|  | yes | 171 | 92.5 % | 83.1 % | 70.3 % |  |
| Forced expiratory volume in 1 second (FEV_1_) in % of the expected value at inclusion into registry (n = 265) | ≤ 50 | 143 | 90.8 % | 79.8 % | 66.7 % | < 0.001 |
|  | > 50 | 122 | 97.7 % | 95.4 % | 92.1 % |  |
| Cardiovascular comorbidity (n = 268) | no | 244 | 93.6 % | 85.5 % | 73.6 % | 0.696 |
|  | yes | 24 | 91.7 % | 83.3 % | 83.3 % |  |
| History of exacerbation at inclusion into registry (n = 157) | no | 101 | 95.1 % | 91.4 % | 91.4 % | 0.004 |
|  | yes | 56 | 82.8 % | 66.8 % | 38.2 % |  |
| Treatment with inhalative antiobstructive agents at inclusion into registry (n = 268) | no | 25 | 100 % | 88.9 % | 76.2 % | 0.545 |
|  | yes | 243 | 92.8 % | 84.8 % | 74.4 % |  |
| Long-term oxygen therapy at inclusion into registry (n = 266) | no | 216 | 94.7 % | 85.8 % | 78.2 % | 0.117 |
|  | yes | 50 | 88.2 % | 81.1 % | 61.8 % |  |
| AAT augmentation therapy at inclusion into registry (n = 268) | no | 157 | 88.2 % | 83.9 % | 81.3 % | 0.991 |
|  | yes | 111 | 98.9 % | 87.9 % | 72.6 % |  |
| Statistical significance (corrected by Bonferroni method): p value < 0.0031  Abbreviations: Alpha-1-Antitrypsin, AAT; Alpha-1-Antitrypsin Deficiency, AATD; body mass index, BMI; chronic obstructive pulmonary disease, COPD; forced expiratory volume in 1 second, FEV_1_ | | | | | | |
