# Supplementary material for: The impact of diagnostic delay on survival in alpha-1-antitrypsin deficiency: results from the Austrian Alpha-1 Lung Registry
Source: Respir Res. 2023 Jan 27;24:34. doi: 10.1186/s12931-023-02338-0 (PMC9881325; doi:10.1186/s12931-023-02338-0)
Supplement: Supplementary file 4 — Additional file 4: Table S4. Multivariable analysis of transplant-free survival (TS) by Cox regression (n = 229). [file 12931_2023_2338_MOESM4_ESM.docx]

| Supplementary table 4. Multivariable analysis of transplant-free survival (TS) by Cox regression (n =229). | | | | |
| --- | --- | --- | --- | --- |
| Variable | | HR | 95 % CI | p value (Cox regression) |
| diagnostic delay (years)* - continuous | | 1.43 | 1.08 - 1.89 | 0.011 |
| Body mass index (BMI; kg/m²) - continuous | | 0.76 | 0.66 - 0.87 | < 0.001 |
| age (years) - continuous | | 1.02 | 0.98 - 1.05 | 0.342 |
| active smoking | no | 1.00 | - | 0.047 |
|  | yes | 3.63 | 1.02 - 12.98 |  |
| Forced expiratory volume in 1 second (FEV_1_) in % of the expected value | > 50 | 1.00 | - | 0.027 |
|  | ≤ 50 | 3.36 | 1.15 - 9.80 |  |
| * after logarithmic transformation  Abbreviations: body mass index, BMI; confidence interval, CI; forced expiratory volume in 1 second, FEV_1_; hazard ratio, HR | | | | |
