# Supplementary figures and images for: The impact of diagnostic delay on survival in alpha-1-antitrypsin deficiency: results from the Austrian Alpha-1 Lung Registry
Source: Respir Res. 2023 Jan 27;24:34. doi: 10.1186/s12931-023-02338-0 (PMC9881325; doi:10.1186/s12931-023-02338-0)

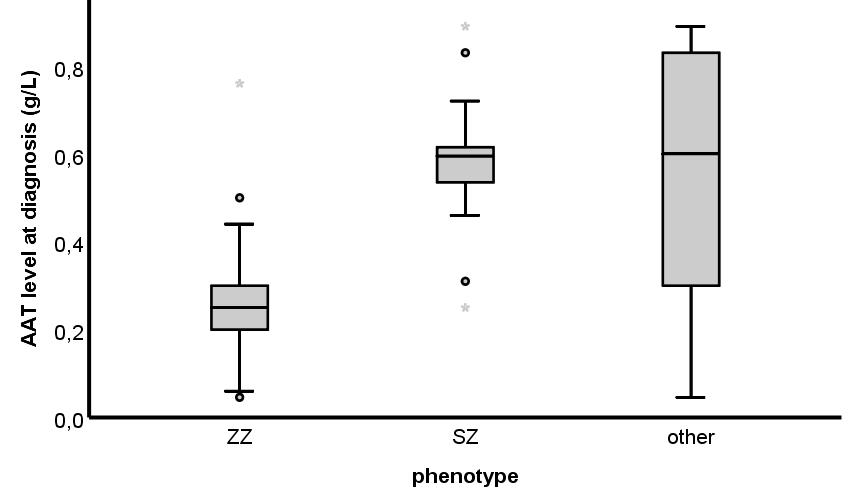

Supplement: Supplementary file 5 — Additional file 5: Figure S1. Boxplot of serum alpha-1-antitrypsin (AAT) level at diagnosis for different phenotypes: Pi*ZZ patients had a significantly lower serum AAT level at diagnosis (mean ± SD: 0.253 ± 0.090 g/L) than Pi*SZ patients (0.584 ± 0.116 g/L; p < 0.001) or patients with other phenotypes (0.532 ± 0.292 g/L; p < 0.001). [file 12931_2023_2338_MOESM5_ESM.jpg]

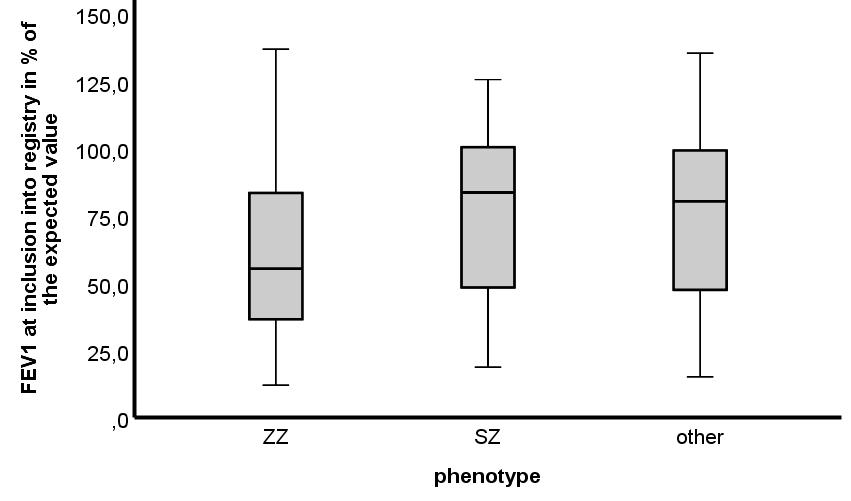

Supplement: Supplementary file 6 — Additional file 6: Figure S2. Boxplot of forced expiratory volume in 1 s (FEV1) in % at inclusion into registry for different phenotypes: FEV1 in % of the expected value at last measurement before inclusion into registry was also significantly lower in Pi*ZZ patients (mean ± SD: 61.4 ± 31.0%) than in Pi*SZ patients (77.8 ± 31.5%; p = 0.003) or patients with other phenotypes (76.1 ± 32.8%; p = 0.007). [file 12931_2023_2338_MOESM6_ESM.jpg]

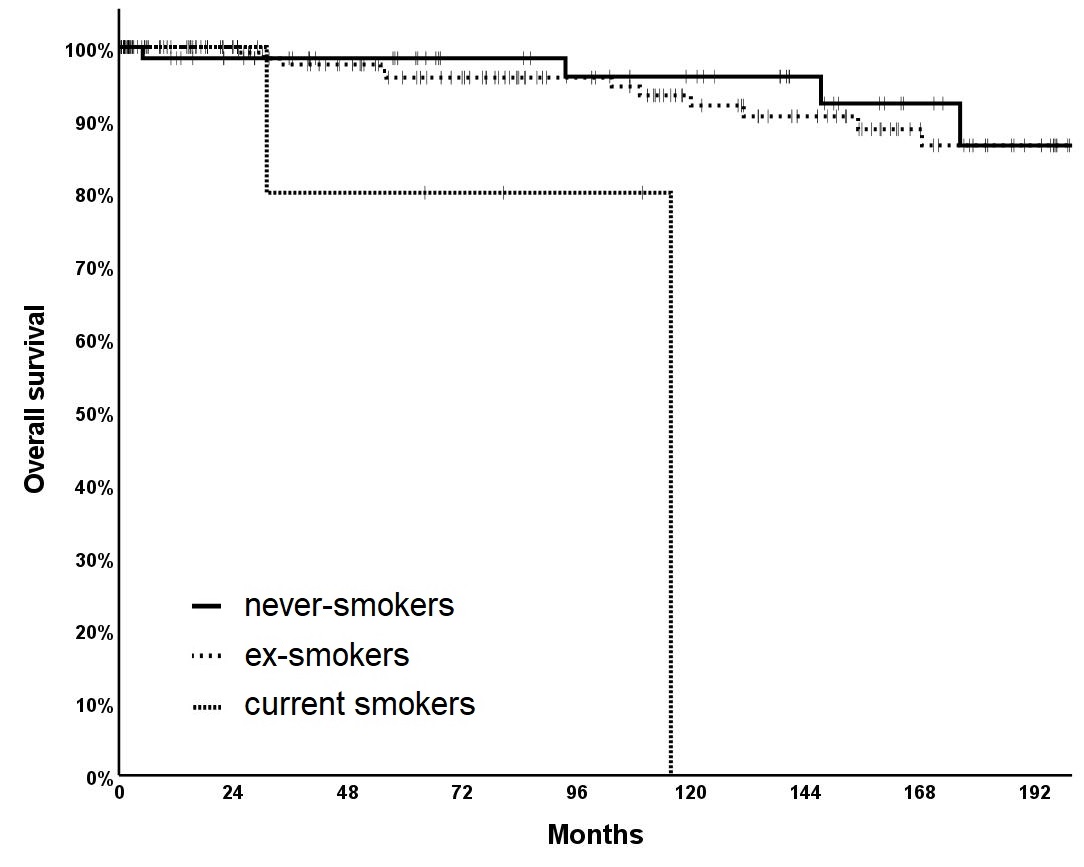

Supplement: Supplementary file 7 — Additional file 7: Figure S3. Kaplan–Meier plot of overall survival (OS) by smoking status at inclusion into registry, never-smokers vs. ex-smokers vs. current smokers (n = 266). [file 12931_2023_2338_MOESM7_ESM.jpg]
